# Visfatin Impairs Endothelium-Dependent Relaxation in Rat and Human Mesenteric Microvessels through Nicotinamide Phosphoribosyltransferase Activity

**DOI:** 10.1371/journal.pone.0027299

**Published:** 2011-11-03

**Authors:** Susana Vallejo, Tania Romacho, Javier Angulo, Laura A. Villalobos, Elena Cercas, Alejandra Leivas, Elena Bermejo, Raffaele Carraro, Carlos F. Sánchez-Ferrer, Concepción Peiró

**Affiliations:** 1 Departamento de Farmacología, Universidad Autónoma de Madrid, Madrid, Spain; 2 Departamento de Histología, Instituto Ramón y Cajal de Investigación Sanitaria (IRYCIS), Madrid, Spain; 3 Servicio de Cirugía General y Digestiva, Hospital de la Princesa, Madrid, Spain; 4 Unidad de Obesidad, Servicio de Endocrinología, Hospital de la Princesa, Madrid, Spain; 5 Departamento de Medicina, Universidad Autónoma de Madrid, Madrid, Spain; 6 Instituto de Investigación Sanitaria del Hospital de la Princesa, Madrid, Spain; University of Padova, Medical School, Italy

## Abstract

Visfatin, also known as extracellular pre–B-cell colony–enhancing factor (PBEF) and nicotinamide phosphoribosyltransferase (Nampt), is an adipocytokine whose circulating levels are enhanced in metabolic disorders, such as type 2 diabetes mellitus and obesity. Circulating visfatin levels have been positively associated with vascular damage and endothelial dysfunction. Here, we investigated the ability of visfatin to directly impair vascular reactivity in mesenteric microvessels from both male Sprague-Dawley rats and patients undergoing non-urgent, non-septic abdominal surgery. The pre-incubation of rat microvessels with visfatin (50 and 100 ng/mL) did not modify the contractile response to noradrenaline (1 pmol/L to 30 µmol/L), as determined using a small vessel myograph. However, visfatin (10 to 100 ng/mL) concentration-dependently impaired the relaxation to acetylcholine (ACh; 100 pmol/L to 3 µmol/L), without interfering with the endothelium-independent relaxation to sodium nitroprusside (1 nmol/L to 3 µmol/L). In both cultured human umbilical vein endothelial cells and rat microvascular preparations, visfatin (50 ng/mL) stimulated nicotinamide adenine dinucleotide phosphate (NADPH) oxidase activity, as determined by lucigenin-derived chemiluminiscence. The relaxation to ACh impaired by visfatin was restored by the NADPH oxidase inhibitor apocynin (10 µmol/L). Additionally, the Nampt inhibitor APO866 (10 mmol/L to 10 µmol/L), but not an insulin receptor-blocking antibody, also prevented the stimulation of NADPH oxidase and the relaxation impairment elicited by visfatin. Accordingly, the product of Nampt activity nicotinamide mononucleotide (100 nmol/L to 1 mmol/L) stimulated endothelial NADPH oxidase activity and concentration-dependently impaired ACh-induced vasorelaxation. In human mesenteric microvessels pre-contracted with 35 mmol/L potassium chloride, the endothelium-dependent vasodilation to bradykinin (1 nmol/L to 3 µmol/L) was equally impaired by visfatin and restored upon co-incubation with APO866. In conclusion, visfatin impairs endothelium-dependent relaxation through a mechanism involving NADPH oxidase stimulation and relying on Nampt enzymatic activity, and therefore arises as a potential new player in the development of endothelial dysfunction.

## Introduction

In 2005, visfatin was identified as a novel adipocytokine preferently released by visceral fat and exhibiting insulin-mimetic properties [1]. This latter statement, however, was retracted due to reproducibility concerns on the hypoglycemic properties of visfatin [2]. Visfatin has been found identical to the cytokine pre-B colony-enhancing factor-1 (PBEF) [3], and to the extracellular form of nicotinamide phosphoribosyltransferase (Nampt), the rate-limiting enzyme in the NAD salvage synthetic pathway biosynthesis that transforms nicotinamide into nicotinamide monoucleotide (NMN) [4].

Recently, several observations have suggested a direct association between plasma visfatin levels and endothelial dysfunction. Thus, in clinical conditions, such as type 2 diabetes mellitus and chronic kidney disease, a positive correlation has been reported between plasma visfatin levels and those of several markers of endothelial injury and systemic inflammation, including soluble vascular cell adhesion molecule-1, intercellular cell adhesion molecule, CD146, monocyte chemoattractant protein, C-reactive protein, or interleukin-6 [5]–[8]. Furthermore, a negative association has been found between circulating visfatin levels and brachial artery flow-mediated dilation (FMD), used as a non-invasive measurement of endothelial function in humans [7], [9], [10]. Indeed, in patients undergoing renal transplantation, visfatin has been identified as a strong predictor of FMD and the reduction of plasma visfatin levels following transplantation is accompanied by the normalization of the endothelial function [11].

The findings presented above allow us for proposing that, besides representing a novel biomarker of endothelial dysfunction, visfatin might also be an active player in directly impairing vascular reactivity. However, little is known at present about the capacity of visfatin to directly influence the vascular tone. To address this question, we used mesenteric arteries from both rat and human origin to explore the influence of visfatin on both contractile and vasorelaxant microvascular responses, with special attention to the role played by two enzymes, namely nicotinamide adenine dinucleotide phosphate (NADPH) oxidase, as a superoxide anions-generating enzyme closely associated to endothelial dysfunction [12], [13], and Nampt.

## Materials and Methods

### Ethics statement

The investigation conforms to the principles outlined in the Declaration of Helsinki and to Spanish legal dispositions. Experiments with human endothelial cells were reviewed and approved by the ethics committee of Universidad Autónoma de Madrid and Hospital Universitario de Getafe. Experiments with human omentum microvessels were reviewed and approved by the ethics committee of Universidad Autónoma de Madrid and Hospital Universitario de La Princesa. In every case, written informed consent was obtained from the donor. The investigation with animals conforms to national guidelines and was approved by the ethics committee of Universidad Autónoma de Madrid (CEI 27–660).

### Materials

Culture plasticware was from TPP (Tragadingen, Switzerland). Culture medium M199, fetal calf serum (FCS) and trypsin-EDTA were from Biological Industries (Beit-Haemek, Israel). Human recombinant visfatin, with an endotoxin level below 0.1 ng per µg, was purchased from Peprotech (London, UK). The blocking mouse monoclonal antibody (Ab-3, clone 47–9) against the insulin receptor (α subunit) was purchased from Labvision (Fremont, CA, USA). The Nampt inhibitor APO866 was kindly donated by Topotarget (Lausanne, Switzerland). Endothelial cell growth supplement (ECGS), noradrenaline (NA), potassium chloride (KCl), acetylcholine (ACh), bradykinin (BK), sodium nitroprusside (SNP), superoxide dismutase (SOD), nicotinamide mononucleotide (NMN), apocynin and, unless otherwise stated, all other reagents were purchased from Sigma Chemical Co. (St. Louis, MO, USA).

### Vascular reactivity

Vascular reactivity was studied in both rat and human mesenteric arteries using a small vessel myograph to measure isometric tension, as previously described [14], [15].

On one side, 3 month-old male Sprague-Dawley rats (250 g) were used for animal studies. Prior to the experiments, the animals were briefly exposed to carbon dioxide in a chamber until they fell unconscious and were then immediately killed by cervical dislocation. The third branch mesenteric arteries (mean internal diameter 309.0±2.5 µm) were dissected, cleaned free of fat and connective tissue, mounted as ring preparations and first contracted with 125 mmol/L KCl followed by washout to determine viability. A first set of experiments was designed to assess the influence of visfatin on NA-induced vasoconstriction. Thus, rat microvascular segments were pre-incubated or not for 20–30 min with visfatin (50 and 100 ng/mL) and then contracted with increasing concentrations of NA (1 pmol/L to 30 µmol/L). A second set of experiments aimed to address the influence of visfatin on endothelium-dependent vasorelaxation. Thus, the microvessels were pre-incubated or not for 20–30 min with visfatin (10 to 100 ng/mL), stimulated with NA (1 µmol/L) and, once the contraction became stable, the vasorelaxant responses to the endothelium-dependent vasodilator ACh (100 pmol/L to 3 µmol/L) were tested by the addition of cumulative concentrations of the drug. The relaxations to SNP (1 nmol/L to 3 µmol/L), as an endothelium-independent vasodilator, were also studied. In some experiments and prior to the contraction with NA, the mesenteric segments were pre-incubated with other compounds, such as NMN, APO866, an insulin receptor blocking antibody, apocynin, L-NAME, indomethacin or SOD, at the indicated concentrations.

The studies in human microvessels were performed using mesenteric arteries obtained from omentum samples from 4 female patients (age, 43±9 yr, range 24–62 yr) undergoing non-urgent, non-septic abdominal surgery at Hospital Universitario de la Princesa, as previously described [15]. After viability confirmation using 125 mmol/L KCl and washout, the human mesenteric segments (mean internal diameter 245.0±2.2 µm) were pre-contracted with 35 mmol/L KCl and subsequently exposed to increasing concentrations of the endothelial-dependent vasodilator BK (1 nmol/L to 3 µmol/L). Some segments were pre-incubated for 20–30 min with visfatin, APO866 or both drugs together at the indicated concentrations before the contraction with 35 mmol/L KCl.

### Endothelial cell isolation and culture

Human umbilical vein endothelial cells (HUVEC) were enzymatically isolated with collagenase and cultured in M199 medium supplemented with 10% FCS, 25 mg/mL ECGS, 100 mg/mL heparin and antibiotics, as previously described [16]. For experiments, HUVEC at passages 1–5 were used.

### NADPH oxidase activity assay

The activity of NADPH oxidase was measured in both HUVEC and rat microvascular preparations by lucigenin-derived chemiluminiscence. Briefly, samples were treated with the different compounds to be tested for 20–30 min. At the end of the treatment, HUVEC were washed in ice-cold phosphate-buffered saline, scraped and centrifuged at 13,000 rpm for 1 minute at 4°C. The resulting cell pellet, as well as the microvascular preparations, was homogenized in lysis buffer (pH 7.0) containing 50 mmol/L KH_2_PO_4_, 1 mmol/L EGTA and 150 mmol/L sucrose at 4°C. For every sample, the protein content was determined by the bicinchoninic acid method. Cellular or microvascular extracts were then incubated in phosphate-buffered saline containing 5 µmol/L lucigenin and 100 µmol/L NADPH and luminiscence was then measured every 10 seconds for 5 minutes in a tube luminometer (Optocomp, MGM Instruments, Hamden, CT, USA). The enzymatic activity was expressed as relative light units (RLU)/µg of protein/min.

### Statistical analysis

Results are expressed as mean±SEM. Statistical analysis was performed using Student's *t* test for data points and ANOVA for curves, with the level of significance chosen at *P*<0.05.

## Results

### Visfatin does not modify NA-induced contraction in rat mesenteric microvessels

**Figure 1:**
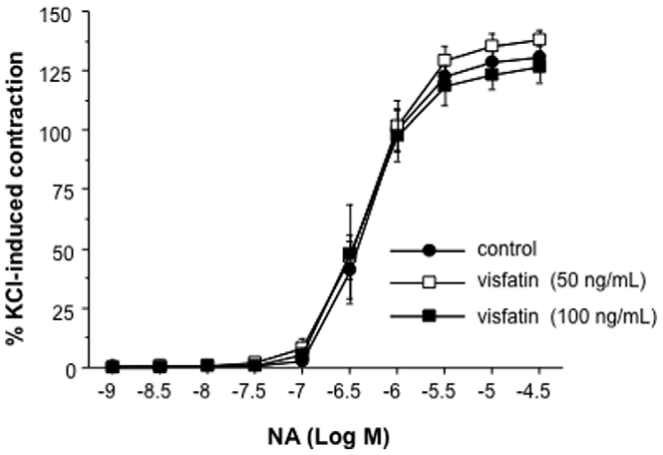
Visfatin does not modify NA-induced contraction in rat mesenteric microvessels. After pre-incubation with visfatin (50 and 100 ng/mL), the microvessels were contracted with increasing concentrations of NA (1 nmol/L to 30 µmol/L). Results are expressed as percentage of a previous contraction elicited by 125 mmol/L potassium chloride (KCl). Results are expressed as mean ± SEM of 17 segments obtained from 4 animals.

In a first set of experiments, the ability of visfatin to modify microvascular contractions was explored. Thus, rat mesenteric arteries, were concentration-dependently contracted with cumulative concentrations of NA (1 nmol/L to 30 µmol/L; Figure 1). The pre-incubation of the microvessels with visfatin (50 and 100 ng/mL) did not modify the contractile responses to NA (Figure 1).

### Visfatin impairs the endothelium-dependent vasodilation to ACh in rat mesenteric microvessels

**Figure 2:**
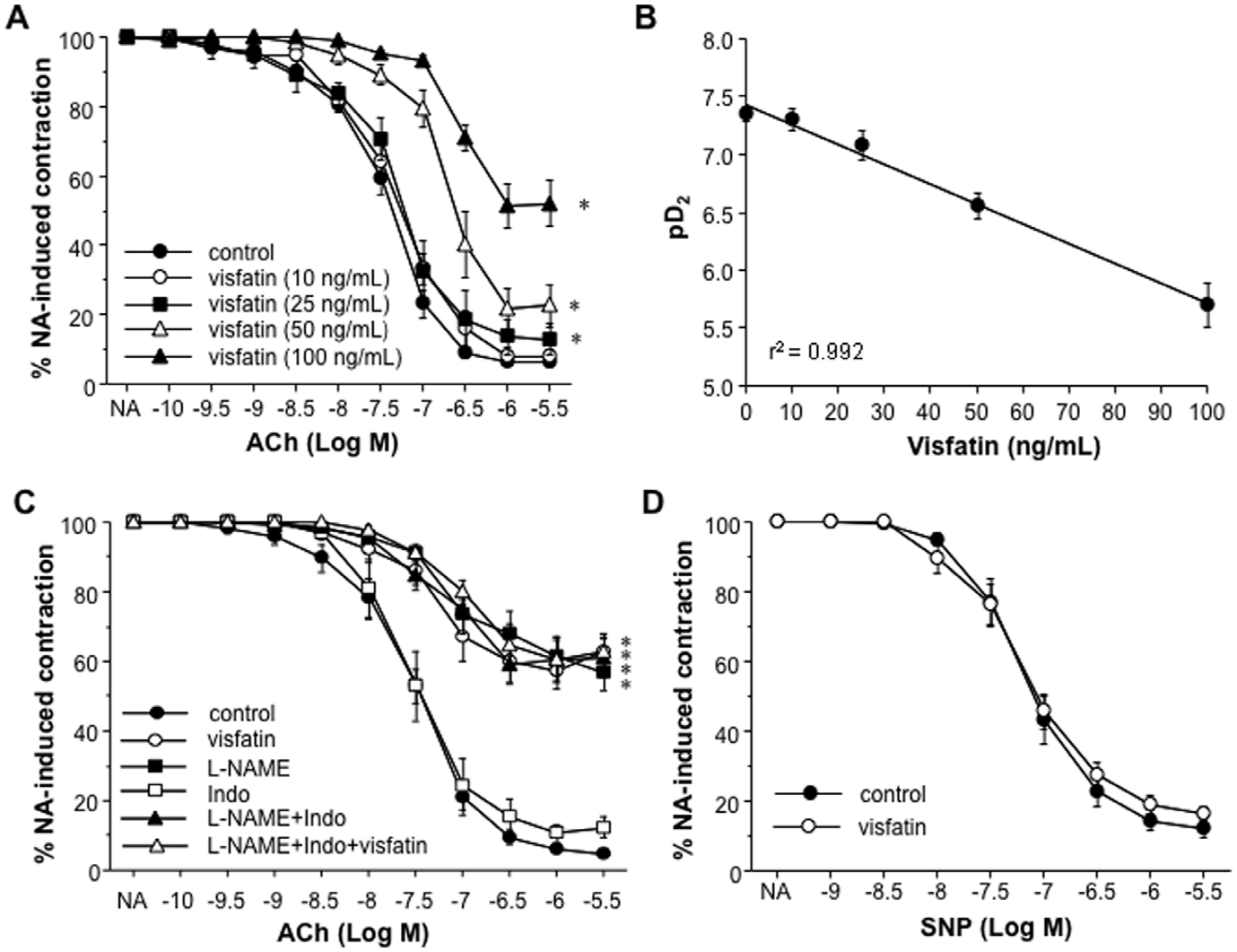
Visfatin impairs the vasodilation induced by ACh in rat mesenteric microvessels. (A) After pre-incubation with visfatin (10 to 100 ng/mL), the microvessels were contracted with 1 µmol/L NA and then exposed to cumulative concentrations of the endothelium-dependent vasodilator ACh (100 pmol/L to 3 µmol/L). Results are expressed as mean ± SEM of 35 segments obtained from 5 animals. *P<0.05 vs control curve. (B) Correlation between the concentrations of visfatin used for pre-incubation and the pD_2_ values for ACh. (C) The microvessels were pre-incubated with visfatin (100 ng/mL), the endothelial oxide synthase inhibitor L-NAME (100 µmol/L), the cyclooxygenase inhibitor indomethacin (Indo; 10 µmol/L) or different combinations of these compounds, and then contracted with 1 µmol/L NA and exposed to cumulative concentrations of ACh (100 pmol/L to 3 µmol/L). Results are expressed as mean ± SEM of 56 segments obtained from 12 animals. *P<0.05 vs control curve. (D) After pre-incubation or not with visfatin (50 ng/mL) and contraction with 1 µmol/L NA, the microvessels were exposed to the endothelium-independent vasorelaxant sodium nitroprusside (SNP; 1 nmol/L to 3 µmol/L). Results are expressed as mean ± SEM of 19 segments obtained from 4 animals.

Despite its lack of effect on NA-induced contraction, visfatin (10 to 100 ng/mL) concentration-dependently impaired the relaxation elicited by cumulative concentrations of ACh (100 pmol/L to 3 µmol/L) in rat microvessels pre-contracted with 1 µmol/L NA (Figure 2A). The correlation between the different concentrations of visfatin used for pre-incubation prior to NA contraction and the pD_2_ values obtained for ACh is shown in Figure 2B.

Furthermore, we observed that the relaxation elicited by ACh in rat microvessels was not modified by indomethacin (10 µmol/L) but was blunted by around 60% in the presence of L-NAME (100 µmol/L) (Figure 2C). Indeed, only the L-NAME-sensitive component of the ACh-induced response was affected by visfatin (100 ng/mL) (Figure 2C).

On the other hand, the pre-incubation of rat microvessels with visfatin (50 and 100 ng/mL) prior to NA contraction did not interfere with the relaxations elicited by the endothelium-independent vasodilator SNP (1 nmol/L to 3 µmol/L) (Figure 2D).

### NADPH oxidase activity mediates the impaired endothelium-dependent vasodilation induced by visfatin in rat microvessels

To gain insight into the mechanisms mediating the impaired endothelium-dependent vasodilation induced by visfatin in rat microvessels, we next explored the impact of the adipokine on NADPH oxidase activation, considered as a major mechanism involved in endothelial dysfunction [12], [13].

**Figure 3:**
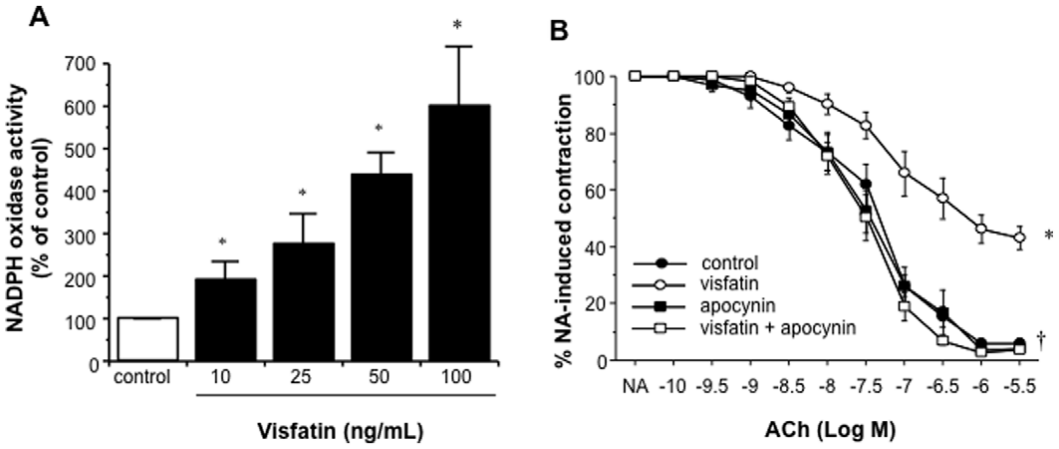
NADPH oxidase mediates the impaired vasodilation to ACh induced by visfatin in rat mesenteric microvessels. (A) Cultured human umbilical vein endothelial cells were stimulated with increasing concentrations of visfatin (10 to 100 ng/mL) for 20–30 min and the generation of superoxide anions by NADPH oxidase was determined lucigenin-derived chemiluminiscence. Results are expressed as mean ± SEM obtained from 6 experiments performed in triplicate. *P<0.05 vs control cultures without visfatin. (B) Rat microvessels were pre-incubated with visfatin (50 ng/mL) and/or apocynin (10 µmol/L), contracted with 1 µmol/L NA and then exposed to cumulative concentrations of ACh (100 pmol/L to 3 µmol/L). Results are expressed as mean ± SEM of 35 segments from 5 animals. *P<0.05 vs control curve; †*P*<0.05 vs visfatin.

As a first approach to address this question, we stimulated cultured HUVEC with increasing concentrations of visfatin (10 to 100 ng/mL) and determined the formation of superoxide anions through NADPH oxidase activity. Thus, after 20–30 min of incubation, visfatin elicited a concentration-dependent activation of endothelial NADPH oxidase activity, as shown in Figure 3A. Similarly, in rat mesenteric microvascular preparations, visfatin, used at a concentration of 50 ng/mL, triggered a marked activation of NADPH oxidase (757,17±13,55% of basal activation, results from three independent experiments; *P*<0,05).

Indeed, the pre-incubation of rat microvessels with the NADPH oxidase inhibitor apocynin (10 µmol/L) totally restored to control levels the impaired relaxation to ACh (100 pmol/L to 3 µmol/L) induced by 50 ng/mL visfatin (Figure 3B). The pre-incubation with apocynin also restored the sensitivity of visfatin-treated microvessels to L-NAME. Thus, the pD_2_ for ACh after pre-incubation with visfatin, apocynin and L-NAME together (7.06±0.17) was equivalent to that obtained after pre-incubation with visfatin plus L-NAME (6.85±0.12) or with L-NAME alone (6.78±0.20), and significantly higher than in control microvessels (7.69±0.16; *P*<0.05; results from 12 segments obtained from 6 animals). In the absence of visfatin, apocynin did not modify the relaxation induced by ACh (Figure 3B).

Furthermore, the co-incubation with the extracellular superoxide anions scavenger enzyme SOD, did not prevent the impaired relaxation to ACh elicited by visfatin (data not shown).

### The activation of NADPH oxidase and the impaired relaxation to ACh induced by visfatin in rat microvessels relies on Nampt activity

**Figure 4:**
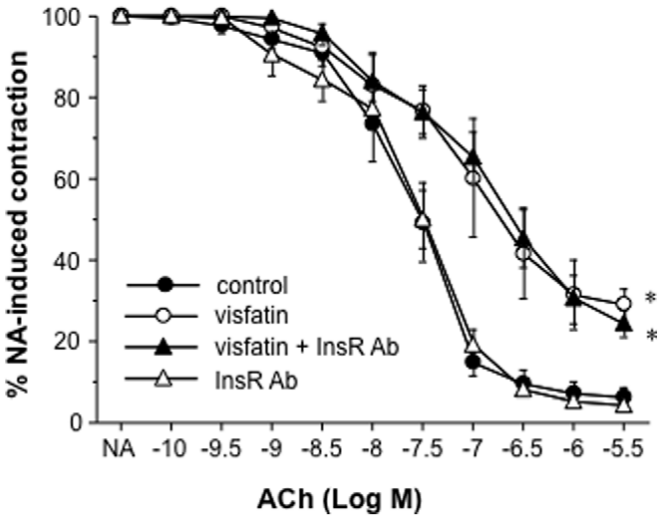
The impaired relaxation to ACh induced by visfatin in rat microvessels does not depend on the insulin receptor. The microvessels were pre-incubated with visfatin (50 ng/mL) alone or combined with either an antibody blocking the activation of the insulin receptor (InsR Ab; 2 µg/mL) and then contracted with 1 µmol/L NA and exposed to increasing concentrations of ACh (100 pmol/L to 3 µmol/L). Results are expressed as mean ± SEM of 20 segments obtained from 4 animals. *P<0.05 vs control curve.

When first described as an adipokine, visfatin was proposed to act through binding to the insulin receptor [1]. Thus, in another set of experiments, the rat microvessels were pre-incubated with a commercial antibody (2 µg/mL) that binds to the α-subunit of the insulin receptor and blocks the receptor activation [17]. Such treatment neither prevented the impairment of ACh-dependent vasodilation induced by visfatin (50 ng/mL) nor modified itself the relaxation to ACh in the absence of visfatin (Figure 4).

**Figure 5:**
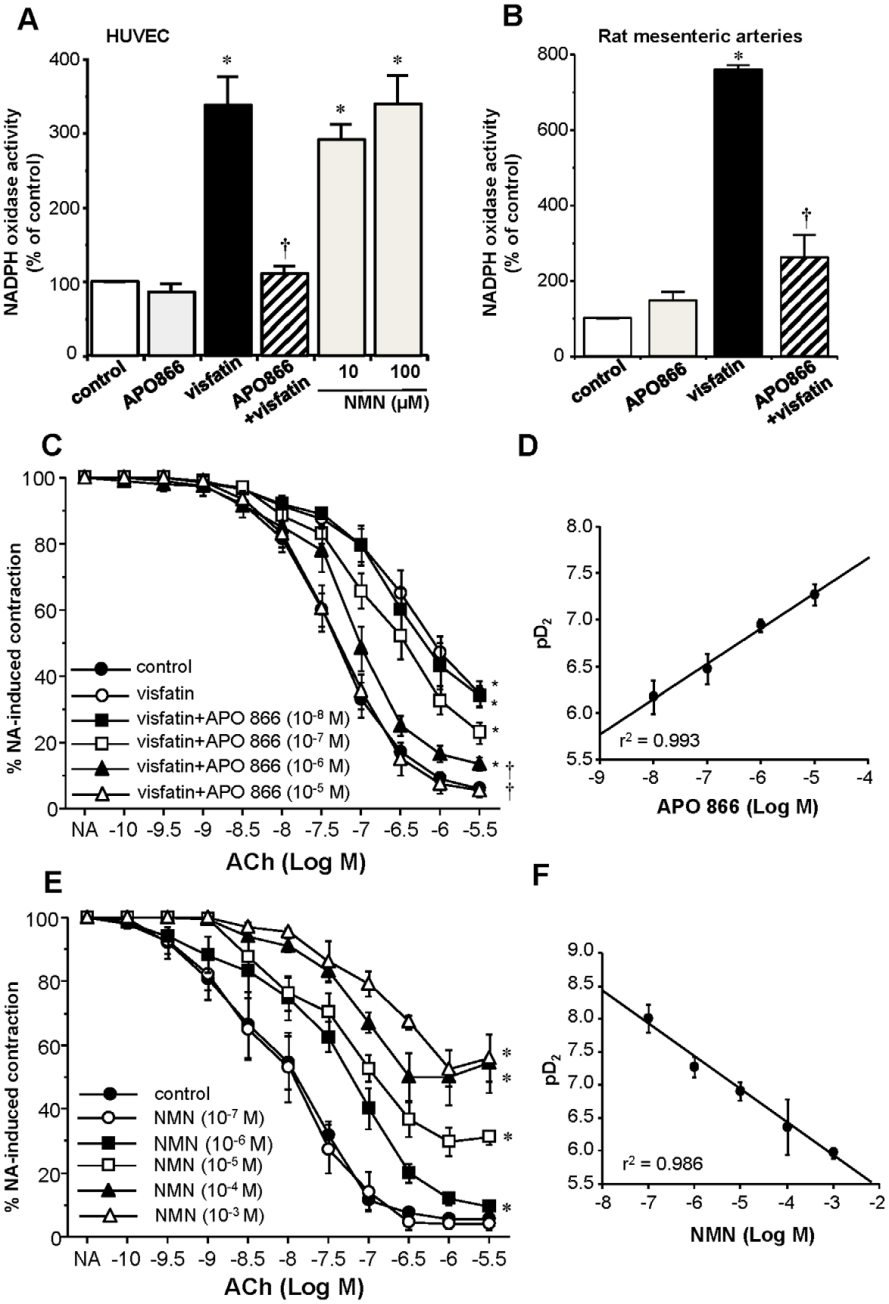
The activation of NADPH oxidase and the impaired relaxation to ACh induced by visfatin in rat microvessels relies on Nampt activity. (A) NADPH oxidase activity was measured in cultured human umbilical vein endothelial cells (HUVEC) stimulated for 20–30 min with visfatin (50 ng/mL), the Nampt inhibitor APO866 (10 µmol/L) or both compounds together, as well as with the product of the Nampt reaction NMN (10 and 100 µmol/L). Results are expressed as mean ± SEM of 5 experiments performed in triplicate. *P<0.05 vs control cultures; †*P*<0.05 vs visfatin (B) Effect of visfatin (50 ng/mL) and APO866 (10 µmol/L), alone or in combination, on NADPH oxidase activity in rat microvascular preparations. Results are expressed as mean ± SEM of 8 segments obtained from 4 animals. *P<0.05 vs control cultures; †*P*<0.05 vs visfatin. (C) Rat microvessels were pre-incubated with visfatin (50 ng/mL), alone or in the presence of APO866 (10 nmol/L to 10 µmol/L), subsequently contracted with 1 µmol/L NA and then exposed to increasing concentrations of ACh (100 pmol/L to 3 µmol/L). Results are expressed as mean ± SEM of 58 segments obtained from 11 animals. *P<0.05 vs control curve; †*P*<0.05 vs visfatin alone. (D) Correlation between the APO866 concentrations used for pre-incubation and the pD_2_ values for ACh. (E) The vessels were pre-incubated with NMN (100 nmol/L to 1 mmol/L), contracted with 1 µmol/L NA and then relaxed with cumulative concentrations of ACh (100 pmol/L to 3 µmol/L). Results represent the mean ± SEM of 32 segments obtained from 5 animals. *P<0.05 vs control curve. (F) Correlation between the NMN concentrations used for pre-incubation and the pD2 values for ACh.

Moreover, we explored the role of Nampt enzymatic activity on the impaired relaxation elicited by visfatin. Thus, the pre-incubation of cultured HUVEC with the Nampt inhibitor APO866 (10 µmol/L) completely prevented the activation of NADPH oxidase triggered by 50 ng/mL visfatin in both HUVEC and mesenteric microvascular preparations (Figures 5A and 5B). Furthermore, the stimulation of HUVEC cultures with the final product of the Nampt reaction NMN (10 and 100 µmol/L) resulted in a marked increase of NADPH activity (Figure 5A). Such effect of NMN (100 µM) was not significantly modified by APO866 (105,99±14,90% of the activation achieved by NMN alone; results from 3 independent experiments performed in triplicate). Moreover, the effect of visfatin (50 ng/ml) on NADPH oxidase activation was not significantly modified by co-incubation with NMN (100 µM) (111,48±8,34% of the activation achieved by visfatin alone in HUVEC; results from 4 independent experiments performed in triplicate).

Similarly, in reactivity experiments, APO866 (10 nmol/L to 10 µmol/L) concentration-dependently restored the impaired microvascular relaxation to ACh (100 pmol/L to 3 µmol/L) elicited by 50 ng/mL visfatin (Figure 5C). Figure 5D depicts the correlation between the APO866 concentrations used and the pD_2_ values for ACh. In the absence of visfatin, APO866 alone did not modify the vascular responses to ACh (data not shown). Moreover, the pre-incubation with NMN (100 nmol/L to 1 mmol/L) impaired in a concentration-dependent manner the vasorelaxant response of rat microvessels to ACh (Figure 5E). The correlation between the NMN concentrations used and the pD_2_ values for ACh is shown in Figure 5F.

### Visfatin impairs the endothelium-dependent vasodilation to BK in human mesenteric microvessels through Nampt activity

**Figure 6:**
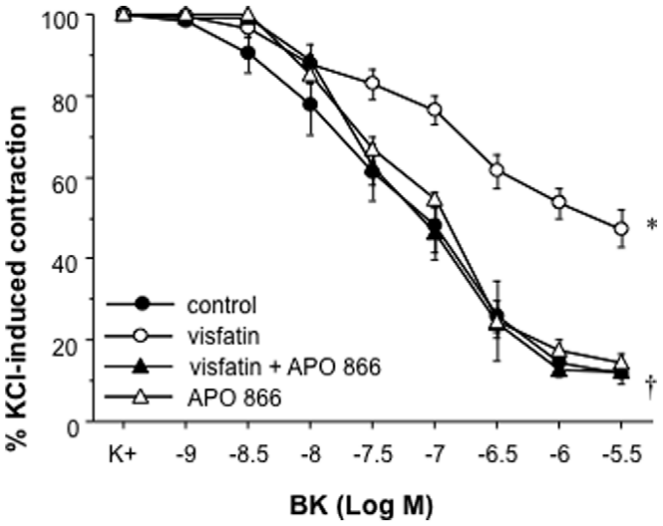
Visfatin impairs the relaxation induced by BK in human mesenteric microvessels via Nampt activity. Human microvessels were pre-incubated with visfatin (50 ng/mL) and/or the Nampt inhibitor APO866 (10 µmol/mL), contracted with 35 mmol/L KCl and then exposed to increasing concentrations of BK (1 nmol/L to 3 µmol/L). *P<0.05 vs control curve; †*p*<0.05 vs visfatin alone. Results are expressed as mean ± SEM of 30 segments obtained from 4 subjects.

We finally assessed whether the impaired vasorelaxation induced by visfatin in rat mesenteric arteries could also be observed in microvessels of human origin. Thus, Figure 6 shows that visfatin (50 ng/mL) impaired the relaxation to BK (1 nmol/L to 3 µmol/L) in human mesenteric arteries. Such an effect was restored by pre-incubation with the Nampt inhibitor APO866 (10 µmol/L), while APO866 itself did not modify the relaxant responses to BK of human microvessels in the absence of visfatin (Figure 6).

## Discussion

Endothelial dysfunction, which can be defined as the loss of balance between the multiple factors controlling reactivity, inflammation, coagulation and cell growth at the vascular level, is a key event tightly associated to the development of vascular diseases, including atherosclerosis and coronary artery disease. In the last years, several clinical studies have shown a positive correlation between enhanced visfatin plasma levels and endothelial dysfunction [5]–[9], while in particular clinical conditions such as renal transplantation, plasma visfatin has been even identified as a strong predictor of FMD both before and after transplantation [11].

In the present study, we have demonstrated that, besides representing a biomarker of endothelial dysfunction and vascular damage, visfatin is also an active player that can directly impair vascular reactivity. More specifically, visfatin reduced the vasorelaxant responses to different endothelium-dependent vasodilators, such as ACh and BK, without affecting the endothelium-independent relaxant capacity of vascular smooth muscle. Importantly, such a deleterious action of visfatin was observed not only in rat microvessels but also in isolated microvessels of human origin. Indeed, visfatin did impair microvascular relaxation from a concentration of 25–50 ng/mL, which can be found in the circulation of patients suffering from obesity, type 2 diabetes or chronic kidney disease, whereas their matched healthy controls exhibit lower levels [11], [18], [19].

To date, the scarce reports available on the regulation of the vascular tone by visfatin have been rather contradictory. Indeed, Yamawaki et al. [20] reported that the addition of visfatin to an organ bath resulted in a modest although significant endothelium-dependent relaxation in both rat aortas and mesenteric arteries pre-contracted with NA. The vasorelaxant action of visfatin appeared to be independent of the insulin receptor, and was explained by the activation of endothelial nitric oxide synthase (eNOS) through phosphorylation at serine 1177 via Akt and de-phosphorylation at threonine 475 [20]. In another *in vitro* study, Lovren et al. [21] further reported the stimulation of both eNOS activity and expression by visfatin, leading to enhanced production of nitric oxide and cyclic GMP formation in cultured human umbilical vein and coronary endothelial cells. However, these effects, which were observed after longer stimulation times with visfatin, were not considered beneficial by the authors, but were rather linked to endothelial cell proliferation and angiogenesis considered as two deleterious pro-atherosclerotic events [21]. More recently, a study by Xia et al. [22] has shown that visfatin inhibits the vasorelaxant response to BK in bovine coronary arteries, which is in line with the results presented herein. Regarding the ability of visfatin to modify the vascular contractility, Yamawaki et al. [20] have reported that visfatin can slightly reduce the vasoconstrictor action of NA in rat aortas. Nevertheless, we could not observe any influence of the adipocytokine on NA-induced vasoconstriction in rat mesenteric arteries.

To gain insight into the mechanisms mediating the impaired vasodilation elicited by visfatin, we explored the potential role of NADPH oxidase. This superoxide anions-generating enzymatic complex has been tightly linked to the development of endothelial dysfunction [12], [13]. Indeed, visfatin rapidly stimulated NADPH oxidase activity in cultured human endothelial cells. Such an acute effect cannot be explained by *de novo* synthesis of NADPH oxidase components, but rather relies on enhanced enzymatic functionality. In this context, it has been recently shown in bovine coronary artery endothelial cells that visfatin favors gp91 subunit aggregation in membrane rafts clusters as well as the translocation of the p47 subunit to these clusters, therefore increasing NADPH activity and superoxide anion release, as determined by electronic spin resonance [22]. Interestingly, visfatin also facilitates NADPH oxidase activation in immune cells, such as human polymorphonuclear neutrophils, by promoting the translocation of p47 and p40 subunits to the cell membrane [23]. In rat mesenteric microvessels, we found NADPH oxidase activity to be a crucial mediator of the impaired vasodilation induced by visfatin through the intracellular release of superoxide anions, since apocynin, but not SOD, prevented such impairment. Although NADPH oxidase can favor endothelial dysfunction through different mechanisms, the quenching and inactivation of endothelial NO by an excess production of reactive oxygen species is a major mechanism linking NADPH oxidase activation and impaired vasodilation [12], [13]. Indeed, in rat mesenteric microvessels, visfatin blunted the vasorelaxant responses sensitive to L-NAME, i.e., dependent on the endothelial release of NO, without affecting a remaining indomethacin- and L-NAME-independent relaxant component, which might be attributed to endothelium-derived hyperpolarizing factor [15]. Furthermore, the fact that rat microvessels pre-incubated with visfatin and apocynin exhibited restored sensitivity to L-NAME further supports a diminished NO bioavailability upon NADPH oxidase activation by visfatin.

As stated before, visfatin was initially claimed to act as insulin mimetic [1]. However, the present study discards a role for insulin receptors in mediating the impaired endothelium-dependent relaxation elicited by visfatin. Instead, Nampt activity arises as the central mechanism mediating the deleterious action of visfatin on microvascular reactivity; thus, the effects of visfatin on NADPH oxidase activation and endothelial-dependent vasodilation were mimicked by NMN, the product of Nampt activity, while it was prevented by the Nampt inhibitor APO866. The fact that visfatin and NMN did not exhibit additive effects on NADPH oxidase activation further suggests that both compounds share a common signaling pathway. In line with these observations, Nampt activity has been shown to mediate several biological actions of visfatin, including vascular smooth muscle cell proliferation and inflammation, the production of matrix metalloproteinases by macrophages, or the release of insulin by β-pancreatic cells [24]–[27].

To date, several reports have demonstrated that visfatin can directly induce a series of vascular events closely related to endothelial dysfunction and vascular damage, such as endothelial cell and vascular smooth muscle cell inflammation [25], [28], [29], vascular smooth muscle proliferation [24], or matrix metalloproteinases activation [27], [30]. In the present study, we provide an additional vasoactive mechanism by which visfatin might further contribute to endothelial dysfunction and to the development of vascular diseases, particularly atherosclerosis. In this context, enhanced circulating visfatin levels have been recently identified as a novel marker of carotid atherosclerosis in type 2 diabetes mellitus patients [31], while a positive association has also been found between plasma visfatin concentrations and CAD, particularly acute coronary syndromes [32]. It is worth noting that a positive correlation also exists between coronary atherosclerosis and the expression of visfatin in the periaortic and pericoronary fat [33], highlighting that not only circulating but also perivascular visfatin might contribute to the onset of endothelial dysfunction and the development of atherosclerotic lesions.

In conclusion, visfatin impairs microvascular endothelium-dependent relaxation through a mechanism involving NADPH oxidase stimulation and relying on Nampt enzymatic activity. Visfatin therefore arises as both a new direct player and a potential therapeutical target in the context of endothelial dysfunction.
